# Intergenerational Transmission of Maternal Adverse Childhood Experiences on Next Generation’s Development: A Mini-Review

**DOI:** 10.3389/fpsyg.2022.852467

**Published:** 2022-05-11

**Authors:** Keita Ishikawa, Natsuko Azuma, Mai Ohka

**Affiliations:** Department of Clinical Psychology, Graduate School of Education, The University of Tokyo, Tokyo, Japan

**Keywords:** adverse childhood experiences, intergenerational transmission, mental health, child development, developmental delay, mini review

## Abstract

(Adverse childhood experiences (ACEs) have extremely harmful impacts on an individual’s physical, social and mental health throughout their life-span. Recently, it has been reported that maternal ACEs increase the risk of developmental delay in the offspring across generations. This mini review focuses on the direct relationship between maternal ACEs and child developmental delay, and potential mediators/moderators that associate their relationship. Six studies were identified using three search engines. The results indicated that four out of six studies reported at least one significant direct association between maternal ACEs and child development. Additionally, maternal biological, psychological, and social factors were identified as mediators or moderators. In summary, we identified that maternal ACEs increased the risk of developmental delay in children *via* biological and psychosocial pathways. Future research should examine potential buffering factors and identify when it is crucial to break the intergenerational transmission.

## Introduction

Studies have shown that adverse childhood experiences (ACEs) have long-term detrimental impacts on physical, social and mental health (Felitti et al., 1998; Anda et al., 2006). According to a recent meta-analysis, exposure to ACEs increases biopsychosocial negative outcomes, such as physical inactivity, diabetes, cancer, smoking, multiple sexual partners, anxiety, depression, and suicide attempts (Hughes et al., 2017). Recently, the scope of ACEs research has extended to the next generation. It has been reported that maternal ACEs increase the risk of offspring’s psychopathology (Cooke et al., 2021; Kang et al., 2021). Besides offspring’s psychopathology, It has been shown that children whose mothers were exposed to ACEs, have a higher risk of having developmental delays (McDonnell and Valentino, 2016). A developmental delay refers to the inability to reach expected milestones in any of in the following developmental domains: gross and fine motor, speech and language, personal and social, and cognitive (Poon et al., 2010). Globally, almost 200 million children under 5 years of age exhibit developmental delays (World Health Organization and World Bank, 2011).

Maternal ACEs can affect offspring’s development influenced by biopsychosocial factors during the perinatal period (prenatal and postnatal). In the prenatal period, Buss et al. (2017) proposed that the main pathway of intergenerational transmission of maternal early life adversity is biological. Adversities in childhood increase the risk of maternal physical health problems during pregnancy, such as dysregulated HPA axis functioning (Bublitz and Stroud, 2012). ACEs are also associated with maternal psychological factors (i.e., depression or PTSD) (Choi and Sikkema, 2016) and social factors (i.e., victimization of Intimate Partner Violence) during pregnancy (Young-Wolff et al., 2019). These maternal psychological and social issues inhibit fetal brain development *via* the maternal intrauterine-placental transmission, increasing offspring’s susceptibility to neurodevelopmental and psychiatric disorders (Buss et al., 2017; Toso et al., 2020). Social and psychological factors were mainly examined as potential variables in the postnatal period. Specifically, substantial evidence has indicated that parenting is a possible variable mediating maternal adversity and child development (Su et al., 2022). Mothers who have experienced childhood adversities are more likely to provide maladaptive parenting to their children because they may have experienced suboptimal care themselves (Bowers and Yehuda, 2016). Furthermore, mothers with a history of ACEs are at a greater risk of postnatal depression or anxiety (Dennis et al., 2019; Letourneau et al., 2019), which reduces their ability to adequately respond to their children’s needs (Bernard et al., 2018). In addition, maternal nurturing skills impeded by adversity have harmful effects on children’s development (Schickedanz et al., 2018; Yoon et al., 2019).

Although a growing body of research has examined the effects of intergenerational transmission of ACEs to the next generation, most studies have focused on offspring’s mental health from a biopsychosocial perspective (Cooke et al., 2021). However, little is known about those effects on offspring’s developmental delay. Therefore this article aims to evaluate (1) the direct association between parental ACEs and offspring’s developmental delay and (2) the various biopsychosocial factors that related to parental ACEs and developmental delay.

## Methods

The authors conducted a literature search through electronic databases (PubMed, PsycArticles, Web of Science) using the following terms: “adverse childhood experience” OR “ACE*” OR “child adversity” AND “intergeneration*” OR “transgenerational cycle.” The literature search included articles published between 1998 and February 6th, 2022, as the first ACE study was published in 1998. The inclusion criteria were as follows: (1) written in English, (2) were empirical research, (3) published in peer-reviewed journals, (4) focus on intergenerational transmission of maternal ACEs and (5) offspring’s outcome were developmental delay. At first, 432 studies were extracted, and 110 duplicates were removed. Next, we excluded studies which (1) were not written in English (*n* = 49), (2)were not empirical research (*n* = 28), (3) were not published in peer-reviewed journals (*n* = 15), (4) were not about intergenerational transmission of maternal ACEs (*n* = 159), (5) offspring’s outcome was not developmental delay (*n* = 65).

## Results

Supplementary Table 1 summarizes the relevant papers. The studies were conducted in three countries [United States (*n* = 4), Canada (*n* = 1), and Taiwan (*n* = 1)]. Three studies targeted low-income parental groups (McDonnell and Valentino, 2016; Sun et al., 2017; Coe et al., 2020).

### Measures of Adverse Childhood Experiences

Of the six articles, four employed the original and most commonly used ACE scale (Felitti et al., 1998). The original scale consists of five items related to maltreatment (physical abuse, sexual abuse, psychological abuse, physical neglect, and psychological neglect) and five items related to family dysfunction (witnessing co-resident mental illness, drug use, arrest, parental divorce, and domestic violence). Based on the original ACE scale, Racine et al. (2018) measured adversities using 11 items across eight categories (emotional abuse, physical abuse, sexual abuse, exposure to familial substance abuse, mental illness, domestic violence, incarceration, and separation/divorce). Chang et al. (2021) used a 14-item revised ACE scale that included four additional adversities: low socioeconomic status (SES), peer victimization, peer isolation/rejection, and exposure to community violence (Finkelhor et al., 2015). All measures of ACEs were reported retrospectively, so there can be a risk of recall bias. Baldwin et al. (2019) found that prospective and retrospective measures of child adversities showed poor agreement, so we need to be careful when interpreting the results.

### Measures of Child Developmental Delay

In all studies, parents responded to validated questionnaires. Three studies used the Age and Stages Questionnaires, Third Edition (ASQ-3;Folger et al., 2018; Racine et al., 2018; Coe et al., 2020), one study used the Age and Stage Questionnaires: Social-Emotional (ASQ-SE; McDonnell and Valentino, 2016), one study used the Parents’ Evaluation of Developmental Status (PEDS; Sun et al., 2017), and one study used the Taiwan Birth Cohort Study–Developmental Instrument (TBCS-DI; Chang et al. (2021). These questionnaires mainly assess the following domains of general development: gross motor, fine motor, problem-solving, communication, and personal/social (Squires et al., 2001; Glascoe, 2003; Singh et al., 2017). The response of these measures was reported by mothers, so the results may contain biases. Adding observation to measurement methods would improve the objectivity of research.

### Maternal Adverse Childhood Experiences and Child Developmental Delay

In this section, we consider whether there is a direct relationship between maternal ACEs and child developmental delay. Table 1 indicates whether there is a direct association between the two main variables.

**TABLE 1:** Direct effect of reviewed studies.

First author, year, country	Direct association
Racine et al. (2018), Canada	×
McDonnell and Valentino (2016), United States	○ (child maltreatment), × (family dysfunction)
Sun et al. (2017), United States	○
Folger et al. (2018), United States	○
Chang et al. (2021), Taiwan	×
Coe et al. (2020), United States	○

*○: means significant effect, × : means insignificant effect.*

To examine the bivariate association between maternal ACEs and child developmental delay, correlation analysis (McDonnell and Valentino, 2016; Racine et al., 2018; Coe et al., 2020; Chang et al., 2021), χ^2^-test (Folger et al., 2018), and crude model of multinomial regression analysis (Sun et al., 2017) were applied. Of the six studies, three showed significant positive bivariate associations between maternal ACEs and child outcomes (McDonnell and Valentino, 2016; Sun et al., 2017; Coe et al., 2020), whereas three showed null results (Folger et al., 2018; Racine et al., 2018; Chang et al., 2021). For example, McDonnell and Valentino (2016) examined the effects of maternal childhood experiences of abuse and family dysfunction on developmental delay in children. The results showed that abuse (not family dysfunction) was positively associated with child developmental delay (*r* = 0.17). Sun et al. (2017) found that mothers who experienced four or more ACEs were more likely to report PEDS concerns (OR = 2.13, 95% CI [1.28, 3.56]). One study that examined the correlation between maternal ACEs and subscales of child development measures indicated that only gross motor skills had a significant positive correlation with maternal ACEs (*r* = 0.15; Coe et al., 2020). In contrast, two studies showed null results (Racine et al., 2018; Chang et al., 2021).

Two studies conducted adjusted analyses and both reported significant positive associations (Sun et al., 2017; Folger et al., 2018). Notably, while Folger et al. (2018) reported no significant effects of maternal ACEs on child developmental delay in bivariate analysis, adjusted analyses (adjusted variables (child sex, race, insurance type, and prematurity) revealed that maternal ACEs were significantly associated with child developmental delay (RR = 1.18, 95% CI [1.08, 1.29]). Unlike Sun et al. (2017) and Folger et al. (2018) reported similar results between bivariate and adjusted analyses, both reporting significant positive effects between maternal ACEs and child developmental delay. In summary, of the six studies, four studies reported at least one significant direct association between maternal ACEs and child development delay (McDonnell and Valentino, 2016; Sun et al., 2017; Folger et al., 2018; Coe et al., 2020), but all studies showed small effect sizes (Cohen, 1992; Chen et al., 2010).

### Mediating or Moderating Pathways

All six studies examined mediating variables, and one study examined a moderating variable. These factors can be divided into three categories; psychological, social, and biological. See Table 2 for details.

**TABLE 2:** Indirect effect of reviewed studies.

	Mediators/Moderators					
	Psychological		Social	Biological		
First author, Year	Prenatal	Postnatal	Postnatal	Prenatal	At birth	Postnatal
Racine (2018)	**Psychosocial risk (< 25 weeks’ gestation)**	Psychosocial risk (4 month)	**Maternal hostile behavior**	**Pregnancy health risk (< 25 weeks’ gestation)**	**Infant health risk**	–
McDonnell and Valentino (2016)	Depression difference score		–	**Maternal age at first pregnancy**	**Infant birth weight**	–
Sun et al. (2017)	–	**Depression**	–	–	–	**Maternal physical health**
Folger et al. (2018)	–	Depression (2 month)	Protective experiences during childhood	–	–	–
Chang et al. (2021)	**Stress, Mental distress (depression and anxiety)**	**Mental distress (depression and anxiety; 6 month)**	–	–	–	–
Coe et al. (2020)	–	–	Maternal scaffolding, maternal sensitivity	–	–	–

*Boldface indicates a significant effect.*

#### Psychological Mediating Pathways

Two studies examined prenatal factors (Racine et al., 2018; Chang et al., 2021), four studies examined postnatal factors (Sun et al., 2017; Folger et al., 2018; Racine et al., 2018; Chang et al., 2021), and one study examined pre to postnatal factors (McDonnell and Valentino, 2016).

As for prenatal factors, all results showed significant results (Racine et al., 2018; Chang et al., 2021). Prenatal psychological risk during pregnancy (social support, history of mental illness, stress, anxiety, and depression) was found to be a significant mediator (Racine et al., 2018). Chang et al. (2021) found that although stress during pregnancy, prenatal mental health (depression and anxiety) did not have mediating effects themselves, three significant pathways linking maternal ACEs to child developmental delay were found: (1) stressful maternal events during pregnancy and maternal postnatal mental health; (2) maternal pre-and postnatal mental health; and (3) maternal stressful events during pregnancy and maternal pre-and postnatal mental health.

As for postnatal factors, two of the four studies reported significant mediating effects (Sun et al., 2017; Chang et al., 2021). Sun et al. (2017) showed that postpartum depression had a significant partial mediating effect. Racine et al. (2018) also reported that maternal postnatal psychosocial risk (measured 4 months after childbirth) did not have an indirect effect. In contrast to the above two studies, Folger et al. (2018) showed that maternal depression (2 months postpartum) did not have an indirect effect on the association between maternal ACEs and child developmental delay.

As for pre to post factors, McDonnell and Valentino (2016) applied a difference score (prenatal depression score minus 6-month depression score) as a psychosocial mediator. Contrary to Racine et al. (2018), the depression difference score did not show an indirect effect on the association between maternal ACEs and child developmental delay (McDonnell and Valentino, 2016).

#### Social Mediating Pathways

Two studies examined mediating effects of maternal parenting. Racine et al. (2018) reported maternal hostile behavior itself did not have an indirect impact; the indirect path from maternal ACEs to child developmental delay *via* maternal prenatal psychosocial risk and subsequent maternal hostile behavior was significant. In contrast, Coe et al. (2020) showed that although the path coefficient from maternal scaffolding to child developmental delay was significant, the path coefficient from maternal ACEs to scaffolding was insignificant. Maternal sensitivity was not significantly associated with maternal ACE or childhood development (Coe et al., 2020).

#### Biological Mediating Pathways

Three studies examined mediating effects of biological factors, and showed significant indirect effects. Racine et al. (2018) found that although maternal pregnancy health risk and infant risk themselves did not have a significant mediating effect, maternal ACEs were associated with poorer child development *via* maternal medical risk and infant risk at birth. McDonnell and Valentino (2016) revealed the age at first pregnancy and infant birth weight, links maternal childhood household dysfunction and child outcome, indicating reproductive health as a potential mediator. Sun et al. (2017) found maternal physical health may be a partial mediator.

#### Moderating Factors

Folger et al. (2018) examined the moderating effect of maternal protective experiences related to individuals, families, and communities during their childhood (e.g., “someone in my family cared about how I was doing in school”). The results showed that the interaction between maternal ACEs and maternal protective experiences was not significant, indicating that in this study, protective experiences did not play a buffering role in the intergenerational transmission of ACEs.

## Discussion

This study aimed to evaluate (1) the direct association between parental ACEs and offspring’s developmental delay and (2) the mediating or moderating effects of various biopsychosocial factors. Regarding direct association (Aim 1), we found that reviewed articles showed inconsistent results (four out of six studies reported significant positive relationships), and the income level of the samples may explain this inconsistency. As for mediating or moderating factors (Aim 2), we found that biopsychosocial factors may be related to the intergenerational transmission of maternal ACEs. Maternal biological and prenatal psychological factors were particularly important. Those two discussions are further explained below (4.1 and 4.2).

### Direct Association Between Maternal Adverse Childhood Experiences and Child Developmental Delay

Previous studies have shown inconsistent results concerning maternal ACEs and child developmental delay. This inconsistency may be explained by the character of the sample. All studies that found significant bivariate association targeted low-income parental groups (McDonnell and Valentino, 2016; Sun et al., 2017; Coe et al., 2020). Although ACEs are experienced by children in all income levels, poverty makes mothers more vulnerable to stress (Hays-Grudo et al., 2021), which may lead to child developmental delay. Further research which focused on mother-child dyads in poverty areas are needed.

### Potential Factors That Explain Intergenerational Transmission of Maternal Adverse Childhood Experiences

Biological, psychological, and social factors have been reported to be mediating or moderating factors. In this section, we indicate the direction for future research by discussing potential buffering factors (“what”) and the crucial time to employ interventions to break the intergenerational transmission of ACEs (“when”).

#### Mediating Pathways

Although the effect sizes for biological processes were small, all studies showed maternal biological factors (prenatal or postnatal) had significant mediating effects (McDonnell and Valentino, 2016; Racine et al., 2018). These results support the hypothesis that intergenerational transmission of maternal ACEs to children’s development occurs during pregnancy *via* stressful environment within maternal uterine-placental environment (Buss et al., 2017). However, to better understand how factors in maternal pregnancy mediate the association between maternal ACEs and child developmental delay, other biomarkers (e.g., the HPA axis) should be examined. Thomas-Argyriou et al. (2021) reported that maternal ACEs were associated with a higher cortisol awakening response (CAR) and flatter diurnal slope (markers of dysregulated HPA axis) during pregnancy, which raised the risk of child psychopathology. The mediating effect of postpartum maternal physical health can be explained by Poppert Cordts et al. (2020), who found that it is associated with lower self-efficacy in parenting, which influences children’s behavioral problems.

Regarding psychological factors, while all studies that examined prenatal mental health reported significant mediating effects, studies that assessed postnatal mental health showed inconsistent results. Racine et al. (2018) and Chang et al. (2021) assessed some aspects of maternal mental health (i.e., depression and anxiety), and reported significant mediating results. Of the three articles that assessed only maternal depression (McDonnell and Valentino, 2016; Sun et al., 2017; Folger et al., 2018), only Sun et al. (2017) reported significant mediating effects. This may be explained by the limited ability of depression to mediate the relationship between maternal ACEs and child development. Folger et al. (2018) considered the reason for not having identified the indirect effect of maternal ACEs on children’s developmental delay to be the absence of other significant measures not captured through a single depression screen. In addition to the various types of maternal mental health, the timing of assessments may also be related to mediating effects. In the reviewed articles, both studies measuring psychological factors during pregnancy revealed significant mediating effects (Racine et al., 2018; Chang et al., 2021). In contrast, of the four studies that measured postnatal maternal mental health (Sun et al., 2017; Folger et al., 2018; Racine et al., 2018; Chang et al., 2021), two studies reported significant mediating effects (Sun et al., 2017; Chang et al., 2021). These results suggest the dominance of prenatal mental health over postnatal health in explaining the intergenerational transmission of ACEs. It is necessary to distinguish the period (prenatal or postnatal) of maternal mental health that contributes to intergenerational transmission. Therefore, longitudinal studies beginning at the onset of pregnancy are required.

Regarding social factors, only two studies have tested the mediating effects of parenting (Racine et al., 2018; Coe et al., 2020). The results suggested that hostile maternal behavior, not scaffolding and sensitivity, had a mediating effect. However, it is difficult to conclude the mediating effect of maternal parenting due to the small number of studies.

Although most coefficients reported in reviewed articles were small (see Supplementary Table 1), variance between maternal ACEs and child developmental delay were also small, indicating that other potential factors which were not assessed in current reviewed articles may be associated with intergenerational transmission.

#### Moderating Factors

One study showed that there was no moderating effect of protective experiences in early childhood, indicating that protective experiences did not play any buffering role in the intergenerational transmission of ACEs. However, in other studies, protective experiences during childhood buffered the negative effect of parental ACEs on harsh parenting attitudes (Morris et al., 2021), depression, PTSD symptoms and stressful life events of pregnant women with ACEs (Chung et al., 2008; Narayan et al., 2018). Narayan et al. (2020) indicated that maternal ACEs and Benevolent Childhood Experiences (BCEs) may have effects on maternal trauma expression and transmission during perinatal period *via* independent pathways. Therefore, further research is needed to determine the buffering role of protective experiences (i.e., BCEs) during parental childhood. To conclude, few studies examined moderating factor, moderator analyses are needed to determine which intervening factors could offset the negative impact of maternal ACEs.

## Conclusion

The limitation of the current study is that only six studies were reviewed, which prevented us from systematically comparing and synthesizing the results of these articles. Although children’s age is associated with how children achieve their developmental milestones, we could not compare the results by grouping children’s age due to scantiness of reviewed articles. It is necessary to increase the number of studies of intergenerational transmission of maternal ACEs and child developmental delay.

## Author Contributions

KI was responsible for the study concept and design. KI acquired data. KI, NA, and MO read the reviewed articles. KI and NA wrote the first draft. MO critically revised the manuscript. All authors analyzed and interpreted the data and read and approved the final manuscript.

## Conflict of Interest

The authors declare that the research was conducted in the absence of any commercial or financial relationships that could be construed as a potential conflict of interest.

## Publisher’s Note

All claims expressed in this article are solely those of the authors and do not necessarily represent those of their affiliated organizations, or those of the publisher, the editors and the reviewers. Any product that may be evaluated in this article, or claim that may be made by its manufacturer, is not guaranteed or endorsed by the publisher.
