# Supplementary material for: Intergenerational Transmission of Maternal Adverse Childhood Experiences on Next Generation’s Development: A Mini-Review
Source: Front Psychol. 2022 May 11;13:852467. doi: 10.3389/fpsyg.2022.852467 (PMC9131025; doi:10.3389/fpsyg.2022.852467)
Supplement: Supplementary file 1 [file Table_1.DOCX]

Supplementary Material

# Supplementary Table

**Supplementary Table 1.** Studies about intergenerational transmission of ACEs on child development

| First author, year | Country | Design, Setting | Sample | Children's age | Measurement of ACEs | Child outcome | Mediator, moderator | Finding |
| --- | --- | --- | --- | --- | --- | --- | --- | --- |
| Folger (2018) | United States | Longitudinal, general | 311 mother-child dyads, 122 father-child dyads | 2 years | The 10-item original ACE scale (Felitti et al., 1998) | Child developmental delay (ASQ-3) | Maternal depression, maternal protective experiences in childhood | In bivariate analysis, both maternal ACEs were not associated with child developmental delay. In adjusted analysis, maternal ACEs were associated with child developmental delay (RR = 1.18, 95% CI [1.08, 1.29]). Maternal postnatal depression did not have a significant mediating effect (p = .78). Interaction between maternal ACEs and maternal protective experiences was nonsignificant (p = .564) |
| McDonnell (2016) | United States | Longitudinal, high risk (low-income) | 398 mother-child dyads | 6 months | The 10-item original ACE scale (Felitti et al., 1998) | Child developmental delay (ASQ-SE; mother report) | Maternal depression (The difference between postnatal score and prenatal score was inputted into SEM), maternal age at first pregnancy, infant birth weight | Maternal family dysfunction was not associated with child developmental delay (r = -.06). Maternal abuse was positively associated with child developmental delay (r = .17). Only the path from maternal family dysfunction through maternal age at first pregnancy and infant birth weight was significant (β = .004, 95% CI [.001, .025]) |
| Coe (2020) | United States | Longitudinal, high risk (low-income) | 295 mother-child dyads | 6, 12 months | The 10-item original ACE scale (Felitti et al., 1998) | Child developmental delay (ASQ-3; mother report) | Maternal scaffolding, maternal sensitivity | Only gross motor skill had significant positive correlation with maternal ACEs (r = .15). Maternal scaffolding and sensitivity did not have indirect effects. |
| Racine (2018) | Canada | Longitudinal, general | 1,994 mother-child dyads | 1 year | The 10-item original ACE scale (Felitti et al., 1998) | Child developmental delay (ASQ-3; mother report) | Pregnancy psychosocial risk, pregnancy health risk, postnatal psychosocial risk, maternal hostile behavior, infant health risk | No significant correlation coefficient between maternal ACEs and children' subscale of ASQ-3. Maternal pregnancy psychosocial risk had a significant mediating effect (β = -.03, 95% CI [-.06, -.01]). Maternal ACEs were associated with poorer child development via maternal medical risk and infant risk at birth (β = -.01, 95% CI [-.01, -.001]). |
| Chang (2021) | Taiwan | Longitudinal, general | 130 mother-child dyads | 6 months | The 14-item revised ACE scale | Child developmental delay (TBCS-DI, 2008; mother report) | Maternal stress during pregnancy, maternal prenatal mental distress (depression and anxiety), maternal postnatal mental distress (depression and anxiety) | No significant correlation coefficient between maternal ACEs and children' subscale of TBCS-DI. Three significant pathways linking maternal ACEs to child developmental delay: 1) through maternal stressful events during pregnancy and maternal postnatal mental health (β = -.03, 95% CI [-.116, -.004]); 2), 2) through maternal pre- and postnatal mental health (β = -.05, 95% CI [-.209, -.011]); 3), 3) through maternal stressful events during pregnancy, and maternal pre- and postnatal mental health (β = -.02, 95% CI [-.114, -.002]). |
| Sun (2017) | United States | Cross-sectional, high risk (low-income) | 1,293 mother-child dyads | 4 months–4 years | The 10-item original ACE scale (Felitti et al., 1998) | Child developmental delay (PEDS; mother report) | Maternal depression, maternal physical health | Unadjusted analysis: 1–3 ACEs increased 1 PEDs (OR = 1.83 (1.18–2.85)) and 2 PEDs concern (OR = 1.72 (1.15–2.56)). ≥4 ACEs increased 1 PEDs (OR=2.13 (1.28–3.56)) and 2 PEDs concern (OR = 1.90 (1.19–3.05)). Adjusted analysis: 1–3 ACEs increased 1 PEDs concern (OR = 1.86 (1.16–3.00)) and 2 PEDs concern (OR = 1.70 (1.07–2.72)). ≥4 ACEs increased 1 PEDs (OR = 2.21 (1.26–3.87)) and 2 PEDs concern (OR = 1.76 (1.02–3.05)). Maternal depression and maternal physical health had significant mediating effects. |

ACEs: Adverse childhood experiences; ASQ-3: Age and Stages Questionnaires, Third Edition; ASQ-SE: Age and Stages Questionnaires, Social-Emotional; TBCS-DI: Taiwan Birth Cohort Study-Developmental Instrument; PEDS: Parents’ Evaluation of Developmental Status
